# CLAVATA signaling in plant–environment interactions

**DOI:** 10.1093/plphys/kiad591

**Published:** 2023-11-01

**Authors:** Sagar Bashyal, Chandan Kumar Gautam, Lena Maria Müller

**Affiliations:** Department of Biology, University of Miami, Coral Gables, FL 33146, USA; Department of Biology, University of Miami, Coral Gables, FL 33146, USA; Department of Biology, University of Miami, Coral Gables, FL 33146, USA

## Abstract

Plants must rapidly and dynamically adapt to changes in their environment. Upon sensing environmental signals, plants convert them into cellular signals, which elicit physiological or developmental changes that allow them to respond to various abiotic and biotic cues. Because plants can be simultaneously exposed to multiple environmental cues, signal integration between plant cells, tissues, and organs is necessary to induce specific responses. Recently, CLAVATA3/EMBRYO SURROUNDING REGION-related (CLE) peptides and their cognate CLAVATA-type receptors received increased attention for their roles in plant–environment interactions. CLE peptides are mobile signaling molecules, many of which are induced by a variety of biotic and abiotic stimuli. Secreted CLE peptides are perceived by receptor complexes on the surface of their target cells, which often include the leucine-rich repeat receptor-like kinase CLAVATA1. Receptor activation then results in cell-type and/or environment-specific responses. This review summarizes our current understanding of the diverse roles of environment-regulated CLE peptides in modulating plant responses to environmental cues. We highlight how CLE signals regulate plant physiology by fine-tuning plant–microbe interactions, nutrient homeostasis, and carbon allocation. Finally, we describe the role of CLAVATA receptors in the perception of environment-induced CLE signals and discuss how diverse CLE-CLAVATA signaling modules may integrate environmental signals with plant physiology and development.

## Teaser

Plants employ signaling peptides and their cognate receptors to dynamically respond to diverse environmental cues.

## Introduction

**Figure 1.**
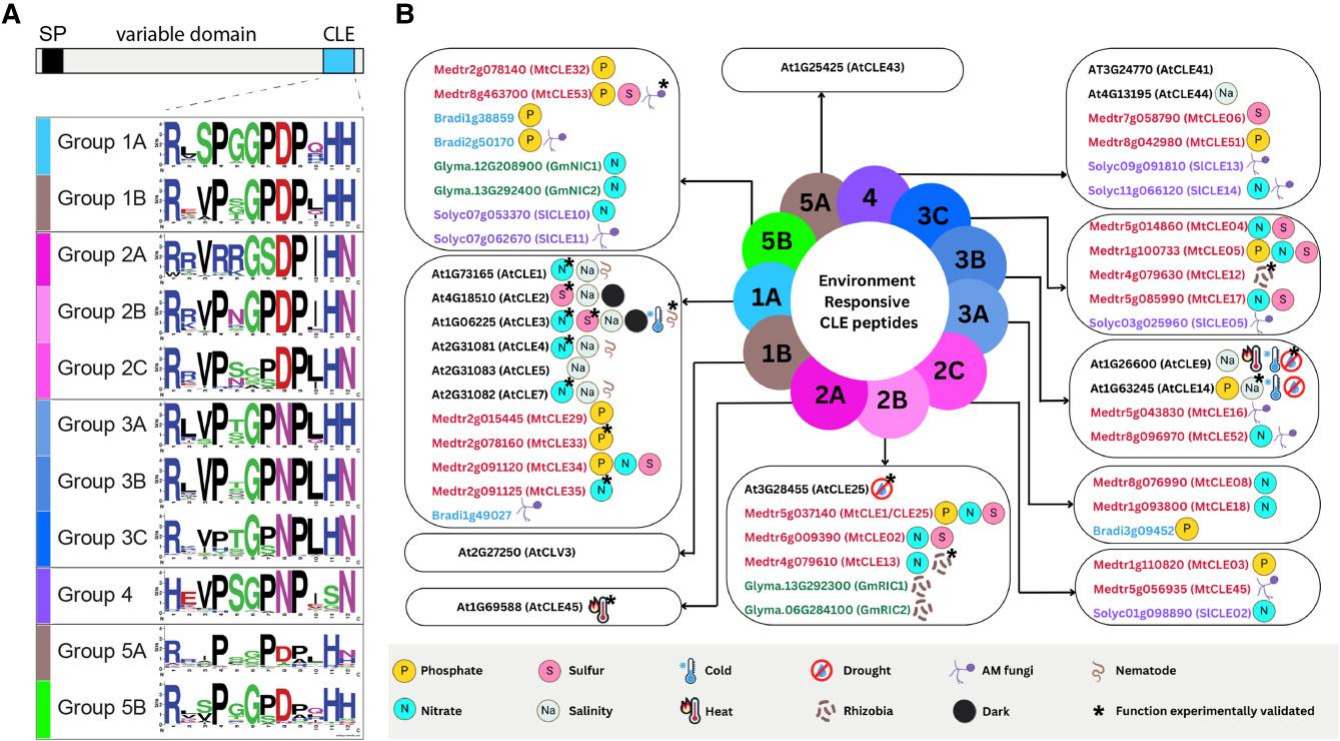
Overview of environment-responsive CLE peptides. **A)** Schematic drawing of a CLE prepropeptide with N-terminal signal peptide (SP), central variable domain, and C-terminal CLE domain (active peptide). Weblogos (Crooks et al. 2004) of CLE peptides show conserved amino acid residues in Groups 1 to 5. CLE groups are based on Zhang et al. (2020). **B)** Transcriptional responsiveness of *CLE* genes from selected species to various environmental stimuli. Groups 1B and 5A do not contain any known *CLE* genes that respond to environmental signals but contain the developmental regulators AtCLV3 and AtCLV43 peptides, respectively. Asterisks indicate experimental evidence for CLE function (see main text for details). *At* (*Arabidopsis thaliana*); *Mt*, *Medtr* (*Medicago truncatula*); *Sl*, *Solyc* (*Solanum lycopersicum*); *Bradi* (*Brachypodium distachyon*); *Gm*, *Glyma* (*Glycine max*). Labels for environmental cues are defined at the bottom of the figure. **B)** was created with Canva.com.

CLAVATA3/EMBRYO SURROUNDING REGION-related (CLE) peptides were initially identified as plant developmental regulators with a critical role in meristem maintenance (reviewed in Fletcher 2020). In brief, CLE peptides are part of negative feedback loops that maintain stem cell numbers in shoot and root apical meristems by balancing cell proliferation and differentiation. Similar CLE-mediated pathways control the differentiation of vascular tissue in the root. Most plant genomes encode dozens of CLE peptides, which are translated in the signaling cell as 50 to 150 amino acid-long prepropeptides with an N-terminal signal peptide, a variable domain, and a highly conserved CLE domain at the C-terminus (Fig. 1A) (Zhang et al. 2020). This 12 to 13 amino acid-long CLE domain, which typically contains hydroxyproline or arabinosylated hydroxyproline residues, represents the active peptide (Ohyama et al. 2009). Posttranslational addition of arabinosyl sugars to hydroxyprolinated residues in the CLE peptide is mediated by glycosyltransferases of the hydroxyproline O-arabinosyltransferase (HPAT) family (Xu et al. 2015).

**Figure 2.**
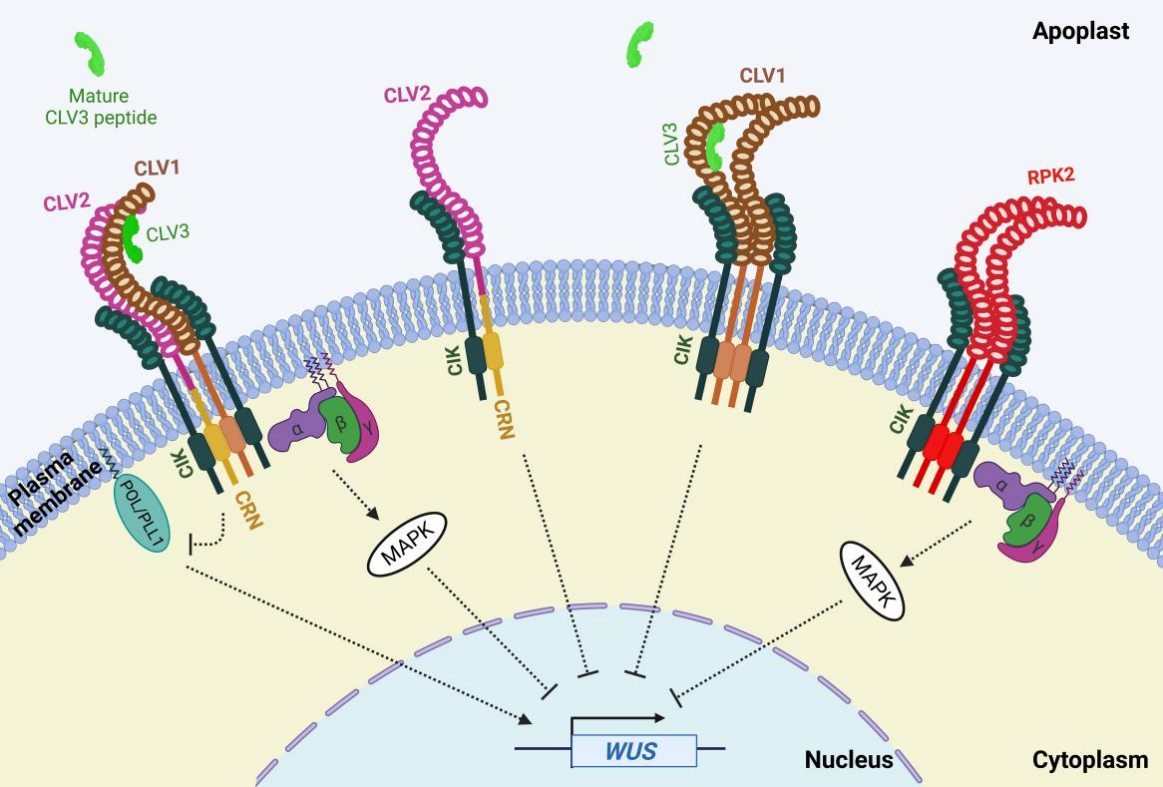
Model illustrating the CLAVATA signaling pathway in the shoot apical meristem. Mature CLV3 peptide in the apoplast binds to the extracellular domains of membrane-associated, homomeric, or heteromeric receptor complexes, which results in the repression of the homeobox transcription factor gene *WUSCHEL* (*WUS*). Signaling also involves interaction between receptor complexes and heterotrimeric G proteins and intermediates like MAPKs and the phosphatases POL and PLL1. Dashed lines indicate putative or indirect signaling pathways. The figure was created with BioRender.com.

Secreted to the extracellular space, mature CLE peptides act as autocrine, paracrine, or endocrine signals (Narasimhan and Simon 2022). Some CLE peptides control physiology and development by transmitting information across cell types, tissues, or organs and are referred to as “peptide hormones” (Roy and Müller 2022). At the target cell, CLE peptides are perceived by plasma membrane–localized receptor complexes consisting of leucine-rich repeat receptor-like kinases (LRR-RLKs) and coreceptors, including but not limited to the LRR-RLK CLAVATA1 (CLV1), the receptor-like protein CLV2, and the membrane-associated pseudokinase CORYNE (CRN), which typically acts in concert with CLV2 (Box 1; Fig. 2) (Somssich et al. 2016). CLAVATA-type receptors have been extensively characterized for their role in shoot and root apical meristem maintenance (Box 1), where CLE binding to the receptor results in the activation of an intracellular signaling cascade involving the protein phosphatases POLTERGEIST (POL) and POLTERGEIST-LIKE1 (PLL1), MAP kinases, and/or the alpha subunit of a heterotrimeric GTP binding protein (Gα) (Fig. 2) (Song et al. 2006; Betsuyaku et al. 2011; Bommert et al. 2013).

**ADVANCES**

- Environmental cues induce specific but partially overlapping sets of *CLE* genes.
- CLAVATA signaling is important for plant developmental adaptations to nutrient availability and abiotic stress.
- CLAVATA signaling is essential for establishing and maintaining symbiotic relationships with rhizobia and AM fungi, as well as defense responses to pathogens.
- Nematode CLE-like peptides hijack CLAVATA-type receptors of plants.
- CLAVATA1 receptors may act as central hubs integrating environmental and developmental cues.
- Bioinformatic tools for identification and phylogenetic clustering of CLE sequences are being developed, which will accelerate functional characterization.

Box 1. **LRR-RLKs in plant growth and development**

Plasma membrane-associated LRR-RLKs are composed of a LRR-containing extracellular domain, a transmembrane domain, and an intracellular kinase domain. LRR-RLKs often form complexes with other LRR-RLKs, LRR receptor-like proteins lacking a kinase domain, and/or membrane-associated kinases lacking a receptor domain (Somssich et al. 2016). Ligands of LRR-RLKs belong to structurally diverse groups, including steroids (brassinosteroids) (Kinoshita et al. 2005) and peptides like phytosulfokine or CLAVATA3 (CLV3)/EMBRYO SURROUNDING REGION (ESR)-related (CLE) peptides (Trotochaud et al. 1999; Matsubayashi et al. 2002). Ligand binding results in the activation of the intracellular kinase domain and subsequent transduction of downstream signaling pathways.

CLAVATA signaling is well understood in the context of shoot apical meristem (SAM) maintenance. Activation of *Arabidopsis* CLAVATA1 (CLV1) by CLV3 binding negatively regulates the homeobox transcription factor WUSCHEL (WUS), required for stem cell identity (Fig. 2). SAM maintenance is also mediated by parallel, CLV1-independent pathways composed of CLV2 and the plasma membrane–localized pseudokinase CRN, the LRR-RLK ERECTA, and/or the LRR receptor RPK2 (Fig. 2); however, CLV3-binding ability has not been reported for these receptors and RLKs (Somssich et al. 2016). In *Arabidopsis*, 3 CLV1-related *BAM* (*BAM1* to *3*) genes are also involved in SAM maintenance (DeYoung et al. 2006). Multiple lines of evidence suggest substantial genetic redundancy and compensation among CLV1-like LRR-RLKs: for example, the *clv1* null mutant phenotype is enhanced by mutations in *BAM* genes (Nimchuk et al. 2015), and *BAM3* reporter gene expression is absent from the center of the SAM in wild-type plants but detectable in strong alleles of *clv1* (Nimchuk 2017). Mutants in *CIKs* are unresponsive to CLV3 peptide treatment and exhibit an enlarged SAM, similar to *clv1* mutants (Hu et al. 2018). Genetic analyses indicate that CIKs mediate CLV3 signaling by acting as coreceptors for CLV1, CLV2/CRN, RPK2, and BAM (Fig. 2) (Anne et al. 2018; Hu et al. 2018; Hu et al. 2022). Although most of our knowledge on CLV1 function comes from studies in *Arabidopsis*, the role of CLV1 in SAM maintenance has been reported to be conserved across vascular and nonvascular plants (Hazak and Hardtke 2016; Mirzaei et al. 2017; Whitewoods et al. 2018). Moreover, CLV1 and related receptors not only regulate SAM maintenance, as distinct CLE-receptor signaling modules regulate lateral root outgrowth, root apical meristem maintenance, and vascular patterning (Fletcher 2020).

Initially, CLE peptides were functionally classified based on their effect on root, shoot, and/or vascular meristems. Application of most *Arabidopsis* (*Arabidopsis thaliana*) CLE peptides caused meristem breakdown and growth arrest; notably, 1 group of CLE peptides did not cause obvious changes in plant development when ectopically applied (AtCLE1 to 7 and AtCLE46) (Hirakawa et al. 2011). Due to their small size and relatively low degree of conservation apart from the CLE domain, the classification of CLE peptides based on sequence similarity is challenging. Recently, sequence-based methods were adopted to cluster CLE prepropeptides (Goad et al. 2017) or the CLE domain (Zhang et al. 2020); the latter approach revealed 5 major bona fide CLE peptide groups based on sequence similarity in 69 plant species (Fig. 1, A and B).

Apart from their role in meristem maintenance, CLAVATA-type receptors and CLE peptides are receiving increased attention for their diverse roles in plant–environment interactions. Here, we first summarize the multiple roles of the CLAVATA signaling pathway in plant responses to abiotic and biotic stimuli. Then, we synthesize how CLE peptides and their cognate receptors may act as central regulators to integrate plant physiological and developmental adaptations to the environment. Finally, we discuss challenges and opportunities for future research on environment-responsive CLAVATA signaling modules.

## CLAVATA signaling modulates plant responses to abiotic factors

Abiotic stress such as drought or extreme temperatures often has a negative impact on plant productivity and can result in decreased agricultural output (Bechtold and Field 2018). In addition, limitations in essential mineral nutrients like nitrogen (N), phosphorus (P), or sulfur (S) affect plant growth and development (White and Brown 2010). Plants respond to abiotic cues through physiological and developmental adaptations. Accumulating evidence suggests that expression of various *CLE* genes changes in response to abiotic environmental stimuli (Fig. 1B). Despite the relatively large number of environment-regulated *CLE* genes, a functional role has only been experimentally validated for some (marked with asterisks in Fig. 1B). Of the environment-induced CLE peptides studied in more detail, some regulate plant developmental adaptations to abiotic factors, such as root system plasticity in response to nutrient availability (Araya et al. 2014; Gutiérrez-Alanís et al. 2017). Conversely, other CLE peptides do not directly affect plant development and instead modulate plant physiological responses like stomatal opening and closing (Takahashi et al. 2018).

### Temperature

Plants have evolved a range of mechanisms to cope with various environmental stressors, including changes in temperature. Plant reproduction is particularly sensitive to heat stress (Zinn et al. 2010).

**Figure 3.**
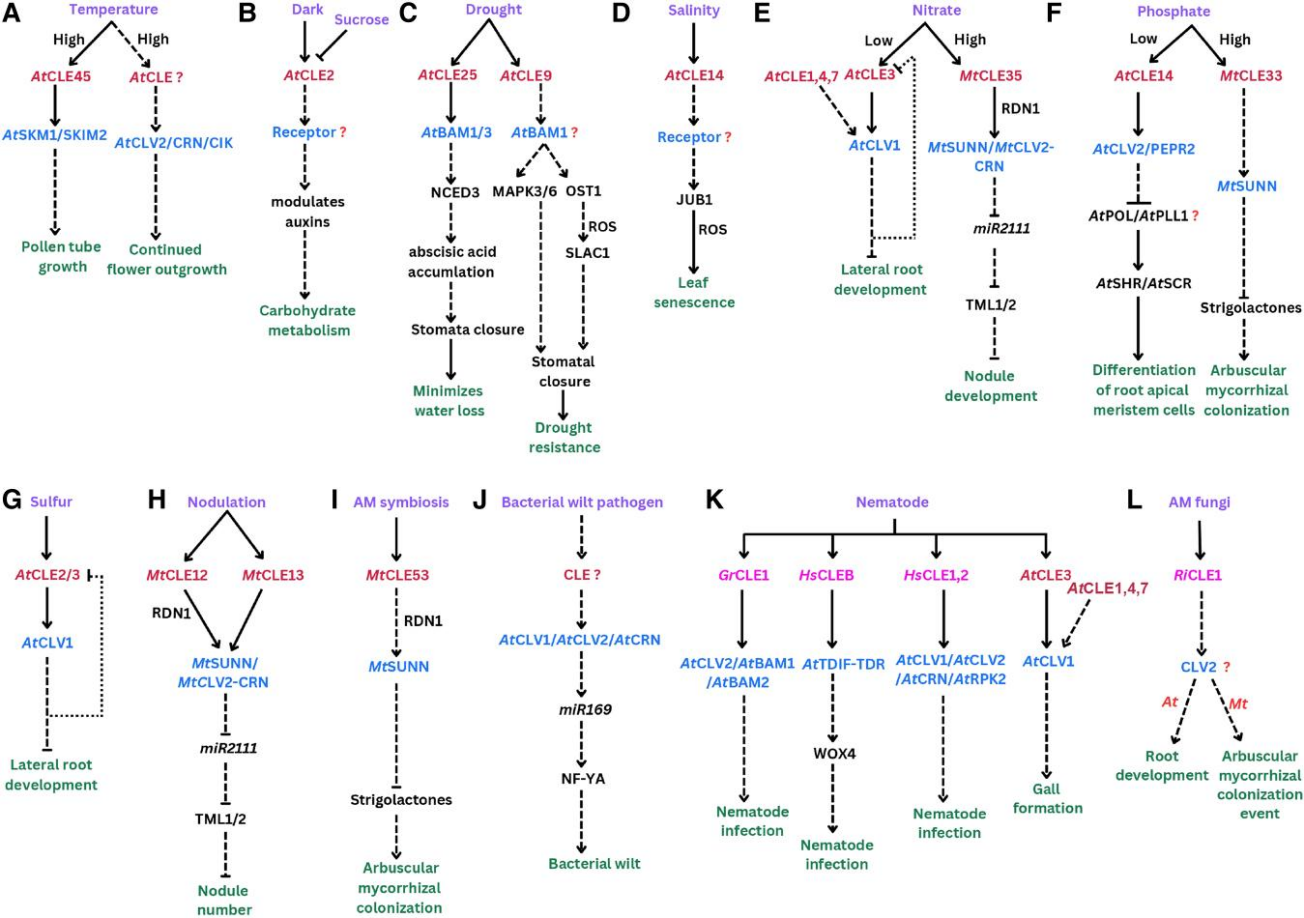
Schematic flowcharts illustrating CLAVATA signaling cascades during plant–environment interactions. **A** to **D)** Abiotic stress-associated CLAVATA signaling in response to temperature, darkness, drought, and salinity, respectively. **E** to **G)** CLAVATA signaling in response to nutrient availability. **H, I)** Plant interactions with mutualistic bacteria (nodulation, **H**) and fungi (AM symbiosis, **I**). **J, K)** Pathogen-associated CLAVATA signaling pathways. Nematodes induce plant *CLE* genes and inject nematode CLE-like peptides, which mimic plant CLE peptides to activate plant CLAVATA signaling **(K)**. It is unknown if the pathway in **J)** is activated by plant or microbial CLE peptides. **L)** AM fungal-derived CLE-like peptides and putative signaling outcomes in planta. **A** to **L)**. Solid lines represent established interactions, and dashed lines indicate putative or indirect signaling mechanisms. Arrows represent activating signals, blunt ends indicate repressive signals. Dotted lines depict negative feedback regulation. Question marks indicate unknown components of the pathways. Figure was created with Canva.com.

In *Arabidopsis*, *AtCLE9 and AtCLE45* are reported to transcriptionally respond to temperature stress (Fig. 1B). Exposure to rising temperatures (shift from 22 °C to 30 °C) expands the expression domain of *AtCLE45* from the stigma to the transmitting tract in the female reproductive organs of wild-type plants. AtCLE45 peptide treatment prolongs pollen tube growth at 30 °C but not at 22 °C (Endo et al. 2013). Pollen tubes defective in the LRR-RLK genes *STERILITY-REGULATING KINASE MEMBER1* (*SKM1*) and *SKM2* are unresponsive to AtCLE45 treatment. Pollen SKM1 can bind AtCLE45, indicating that the AtCLE45-SKM pathway plays a crucial role in successful seed production under high-temperature conditions (Fig. 3A; Endo et al. 2013). On the other hand, the functional role for AtCLE9 in plant temperature responses remains unclear; expression of this gene is also induced under drought conditions (discussed below), suggesting that heat and drought affect similar CLE signaling pathways.

Recently, the *Arabidopsis* receptor/pseudokinase pair AtCLV2/AtCRN and CLAVATA3 INSENSITIVE RECEPTOR KINASE (AtCIK) coreceptors were implicated in controlling temperature-dependent continued flower outgrowth, a process that is critical for reproductive success (Jones et al. 2021): under ambient temperatures, flower primordia in certain *clv2*, *crn*, and *clv3-insensitive kinase 1/2/4* (*cik1/2/4*) mutant alleles terminate early, indicating that a putative CLV2/CRN/CIK receptor module and its yet unknown ligand are involved in sustained flowering under these conditions (Fig. 3A). Interestingly, when the same mutants were grown at high temperatures, early termination of flowering was not observed. This suggests that at high temperatures, the relative importance of CLV2/CRN/CIK signaling for sustained flowering decreases. Instead, a parallel pathway is initiated that promotes flowering through heat-induced auxin biosynthesis (Jones et al. 2021). Although it is unknown which CLE peptides are involved in temperature-dependent CLV2/CRN/CIK signaling, these findings indicate that the CLAVATA pathway is part of an environmental buffering mechanism ensuring reproductive success (Jones et al. 2021).

Temperature-dependent *CLE* expression was also studied in multiple other plant species: elevated temperature resulted in upregulation of apple (*Malus domestica*) *MdCLE6*/*21* and *MdCLE18* and grape vine (*Vitis vinifera*) *VvCLE6* (Wang et al. 2019; Zhang, Li, et al. 2022). The functional roles of these peptides have not been examined, but further exploration may prove useful for producing heat-resilient crops.

Exposure to cold conditions (4 °C) in *Arabidopsis* seedlings also leads to differential regulation of several *CLE* genes (Fig. 1B) (Wang et al. 2016; Ma et al. 2020; Zhang, Liu, et al. 2022). Cold stress increased expression level of *MdCLE6*/*21*, *MdCLE10*, and *MdCLE16*, and decreased *MdCLE17* expression in apple seedlings (Zhang, Li, et al. 2022). The functional role of these CLE peptides has not been investigated. Nevertheless, these findings suggest susceptibility of *CLE* gene expression to thermal fluctuations, highlighting the importance of considering the impact of temperature when studying CLE signaling pathways.

### Light/dark and carbon

Light is a critical factor that regulates plant growth (Liu et al. 2017). Besides providing energy for photosynthesis, light also serves as an essential environmental cue that regulates various physiological processes in plants. One of these processes is the activation of genes involved in light-dependent carbohydrate metabolism, which is crucial for plant growth and survival (Rodriguez-Aparicio et al. 1987).

A recent study sheds light on the role of CLE signaling in light-dependent carbohydrate metabolism in *Arabidopsis* seedlings (Fig. 3B) (Ma et al. 2020): under dark conditions, *AtCLE2* and *AtCLE3* gene expression is induced in the root vasculature (Fig. 1B). Sucrose application to roots suppressed this upregulation; furthermore, *cle2 cle3* double mutants accumulated less sucrose and glucose in their roots than wild types, suggesting that *AtCLE2* and *AtCLE3* are signals for low root carbon status and act as positive regulators of root carbon accumulation (Okamoto et al. 2022). *AtCLE2* overexpression in roots suppresses lateral root growth likely through intersection with auxin signaling (Araya et al. 2014; Ma et al. 2020), indicating that CLAVATA signaling allows plants to restrict energy-consuming developmental processes under low carbon conditions. Interestingly, estradiol-induced *AtCLE2* expression in roots also resulted in differential expression of carbohydrate metabolism–associated genes in shoots (Ma et al. 2020). Mass spectrometry analyses of *Arabidopsis* xylem sap revealed that AtCLE2 moves from the root to the shoot (Okamoto et al. 2022), suggesting that the peptide may be part of a systemic signaling pathway that integrates carbon availability with plant growth and development.

### Drought

Drought stress has profound impacts on plant productivity, leading to—among other effects—loss of turgor pressure, reduced photosynthesis caused by stomatal closure, altered carbon partitioning, and overall reduced growth (Farooq et al. 2009). Studies in *Arabidopsis* and apple seedlings revealed that drought treatment results in expression level changes of several *CLE* genes, including *AtCLE9*, *AtCLE25*, and *MdCLE4*/*5* (Takahashi et al. 2018; Zhang et al. 2019; Zhang, Li, et al. 2022) (Fig. 1B). When *Arabidopsis* roots detect a decrease in water potential, transcription of *AtCLE25* is activated (Takahashi et al. 2018). AtCLE25 is then transported through the vascular system to the leaves, where it binds to BARELY ANY MERISTEM (BAM) receptors (Takahashi et al. 2018). This association triggers a signaling pathway that leads to an increase in the expression of NINECISEPOXY-CAROTENOID DIOXYGENASE3 (NCED3), an enzyme involved in the biosynthesis of abscisic acid (ABA). ABA accumulation results in stomata closure and minimizes water loss through transpiration, indicating that AtCLE25 is part of a systemic signaling pathway modulating shoot physiological adaptations in response to root water status (Fig. 3C) (Takahashi et al. 2018). Another drought-induced *CLE*, *AtCLE9*, is expressed in stomata. AtCLE9 peptide application or overexpression induces closure of stomatal pores in leaves, which resulted in enhanced plant resistance to drought stress (Zhang et al. 2019). Although the receptor remains unknown, the signaling cascade downstream of AtCLE9 is relatively well understood and involves the MITOGEN-ACTIVATED PROTEIN KINASE3 (MAPK3)/MAPK6, OPEN STOMATA1 (OST1), SLOW ANION CHANNEL-ASSOCIATED1 (SLAC1), and reactive oxygen species (ROS) (Zhang et al. 2019) (Fig. 3C).

In addition to their functions in plant drought response, both AtCLE9 and AtCLE25 also have roles during plant development: AtCLE9 mediates the division of the stomatal lineage in the shoot and the division of xylem precursor cells in the root (Qian et al. 2018). These mechanisms were reported to be dependent on the HAESA-LIKE 1 (HSL1)-SOMATIC EMBRYOGENESIS RECEPTOR KINASE (SERK) receptor complex and AtBAM1, respectively (Qian et al. 2018). Notably, the role of receptors in tissue-specific AtCLE9 signaling requires further study: while AtCLE9-AtBAM1 binding was experimentally verified, structural analysis of HSL1-CLE9-SERK1 complex suggested that AtCLE9 is only partially recognized by HSL1 (Roman et al. 2022). In addition to its systemic role in drought response, AtCLE25 mediates local phloem pole pattering in concert with other peptides (AtCLE26 and AtCLE45) and receptors, including BAMs, CIKs, RECEPTOR-LIKE PROTEIN KINASE 2 (RPK2), and CLE-RESISTANT RECEPTOR KINASE (CLERK) (Ren et al. 2019; Gujas et al. 2020; Hu et al. 2022; Qian et al. 2022; Diaz-Ardila et al. 2023). It is unclear if the receptors associated with the developmental roles of AtCLE9 and AtCLE25 are also required for their function in drought response signaling. Further research is needed to fully understand the complex signaling pathways involved in plant responses to changes in water availability and intersection with developmental signaling, including the roles of receptors.

### Salinity

Excess soil salinity poses a substantial threat to crop productivity by causing metabolic damage and osmotic stress, which can ultimately result in plant death (Isayenkov 2012). Salinity stress also results in plant developmental adaptations, including remodeling of the root system to reduce exposure to salt (Zou et al. 2022).

Salt treatment affects the expression of the soybean (*Glycine max*) *CLV1* ortholog *NODULE AUTOREGULATION RECEPTOR KINASE* (*NARK*) (Cheng et al. 2018; Table 1) and several *CLE* genes in different plant species (Fig. 1B): *Arabidopsis* NaCl-regulated *CLE* genes include *AtCLE5*, *AtCLE9*, *AtCLE14*, and *AtCLE44*, all of which are upregulated upon salt stress (Wang et al. 2016; Zhang et al. 2019; Ma et al. 2020; Zhang, Liu, et al. 2022). As noted above, *AtCLE9* and *AtCLE14* are also regulated by drought stress (Wang et al. 2016; Zhang, Liu, et al. 2022); a reason for this could be that salinity causes osmotic stress. A recent study reported minor sensitivity of apple *CLE* genes upon NaCl treatment, but several were reported to be slightly upregulated or downregulated (Zhang, Li, et al. 2022).

**Table 1.** Distinct roles of CLAVATA1 receptors in plant–environment interactions and development across species

CLAVATA1 LRR-RLK	Function	CLE peptide	Co-receptors	Downstream signaling	References
*Arabidopsis thaliana* CLAVATA1 (AtCLV1)	N-dependent inhibition of lateral root outgrowth	CLE1/3/4/7			Araya et al. (2014)
	Inhibition of lateral root development under sulfur deficiency	CLE2/3			Dong et al. (2019)
	Resistance to oomycetes, fungi, and bacterial pathogens		CLV2, CRN	*miR169*, NF-YA	Hanemian et al. (2016)
	Perception of nematode CLE-like peptides	*Heterodera schachtii* CLE	CLV2, CRN, RPK2	WOX4	Replogle et al. (2011, 2013); Guo et al. (2017)
	Root-knot nematode gall formation	CLE3			Nakagami et al. (2023)
	Shoot apical meristem maintenance	CLAVATA3	CLV2, BAM, RPK2, CIK, CLV2-CRN	POL, PLL1, MAPK, WUSCHEL	Leyser and Furner (1992); Zhu et al. (2010); Hu et al. (2018)
	Root apical meristem maintenance	CLE40	ARABIDOPSIS CRINKLY4 (ACR4)	WOX5	Berckmans et al. (2020)
*Brachypodium distachyon* FLORAL ORGAN NUMBER1 (BdFON1)	Regulation of AM symbiosis				Müller et al. (2019)
*Glycine max* nodule autoregulation receptor kinase (GmNARK) and GmCLV1A/B	Response to abiotic (ABA and NaCl) stresses (GmNARK)				Cheng et al. (2018)
	Regulation of AM symbiosis (GmNARK)				Meixner et al. (2005); Schaarschmidt et al. (2013)
	Long-distance communication with nodule and lateral root primordia (GmNARK)	RIC1, RIC2			Searle et al. (2003); Lim et al. (2011)
	Nitrate and rhizobium induced regulation of nodulation (GmNARK)	RIC1, RIC2, NIC1			Reid et al. (2011)
	Flower, leaf, and stem development (GmCLV1A)				Mirzaei et al. (2017)
*Pisum sativum* symbiosis 29 (PsSYM29)	Autoregulation of nodulation		CLV2		Krusell et al. (2002); Morandi et al. (2000)
	Regulation of AM symbiosis				
*Phaseolus vulgaris* nodule autoregulation receptor kinase (PvNARK)	Autoregulation of nodulation	RIC1			Ferguson et al. (2014)
*Medicago truncatula* supernumerary nodules (MtSUNN)	Regulation of nodulation and root length	CLE12, CLE13	CRN		Schnabel et al. (2005); Kassaw et al. (2017); Nowak et al. (2019)
	N-dependent inhibition of nodulation	CLE35		*miR2111*	Moreau et al. (2021); Mens et al. (2021)
	N-dependent inhibition of lateral root outgrowth			represses shoot-to-root auxin transport	Jin et al. (2012)
	Autoregulation and P-regulation of AM symbiosis	CLE53, CLE33			Karlo et al. (2020); Müller et al. (2019)
*Lotus japonicus* hypernodulation aberrant root (LjHAR1/SYM78)	Shoot-controlled regulation of root growth, nodule number, and N inhibition of nodulation	CLE-RS1, CLE- RS2	CLV2, KLAVIER	cytokinin, *miR2111, TML*	Krusell et al. (2002); Okamoto et al. (2009); Sazaki et al. (2014); Tsikou et al. (2018); Solaiman et al. (2000)
	Regulation of AM symbiosis				
*Solanum lycopersicum* fasciated and branched (SlFAB)	Regulation of shoot meristem activity and inflorescence architecture				Xu et al. (2015)
	Regulation of AM symbiosis		CLV2		Wang, Velandia, et al. (2021)

Salinity-induced AtCLE14 accumulation was shown to regulate leaf senescence by controlling the homeostasis of ROS (Zhang, Liu, et al. 2022). Leaf senescence is influenced by endogenous (e.g. phytohormones and age) and environmental cues (e.g. abiotic or biotic stress) (Huang et al. 2022). *Arabidopsis* leaf senescence was delayed upon *AtCLE14* overexpression or treatment with synthetic peptide, whereas *cle14* knockout mutants exhibited accelerated progression of normal and salinity-induced leaf senescence (Zhang, Liu, et al. 2022). AtCLE14 signaling reduces ROS levels through activation of ROS scavenging genes by the NAC family transcription factor JUNGBRUNNEN1 (JUB1) and is therefore suggested to act as a “brake signal” during leaf senescence (Fig. 3D) (Zhang, Liu, et al. 2022).

NaCl-induced AtCLE44 has been previously associated with a function in root development: like the closely related *AtCLE41*, *AtCLE44* encodes a CLE peptide also referred to as TRACHEARY ELEMENT DIFFERENTIATION INHIBITORY FACTOR (TDIF), which is perceived by the LRR-RLK TDIF RECEPTOR/PHLOEM INTERCALATED WITH XYLEM (TDR/PXY) (Li et al. 2017). TDR/PXY shares 42% of sequence identity with CLV1, and the TDIF/PXY module is known to mediate root vascular tissue patterning (Li et al. 2017; Bagdassarian et al. 2020). Because salt treatment influences root development, it is unclear if *AtCLE44* induction by NaCl is a cause or a consequence of salinity stress–mediated root developmental changes in plants.

### Nitrogen

Plants exhibit remarkable root system plasticity in response to N availability: primary root growth and lateral root development are inhibited when N is absent or when plant N status is high, whereas low or heterogeneously distributed N stimulates lateral root growth (Jia and von Wirén 2020). Shoot plasticity in terms of branching pattern and rosette size has also been observed in response to varying N levels (de Jong et al. 2019; Duarte et al. 2021).

Many N-regulated CLE peptides have been studied in the context of plant symbioses with beneficial microbes (discussed below). However, the CLAVATA pathway also plays a role in regulating symbiosis-independent plant responses to N: in *Arabidopsis*, the root vasculature accumulates a signaling module made up of N-responsive CLE signaling peptides (AtCLE1, AtCLE3, AtCLE4, and AtCLE7) and the LRR-RLK CLV1, which regulate lateral root development in an N-dependent manner (Figs. 1B and 3E) (Araya et al. 2014): *AtCLE1/3/4/7* are expressed in pericycle cells in response to N deficiency, and overexpression of these *CLE* genes inhibited the outgrowth (but not the initiation) of lateral roots (Araya et al. 2014). Conversely, *clv1* mutants displayed continuous lateral root emergence from the primary root even in N starving conditions, resulting in more and longer lateral roots in the mutant relative to the wild type; *clv1* mutants also exhibited an overaccumulation of *AtCLE2/3/4/7* transcripts, indicating feedback regulation of these *CLE* genes by the CLV1 receptor (Araya et al. 2014).

A N-responsive CLE-CLAVATA1 module appears to also regulate root system architecture in some legumes: mutants in the CLV1 orthologs of *Medicago truncatula* (*SUPERNUMERARY NODULES*, *MtSUNN*), *Lotus japonicus* (*HYPERNODULATION ABERRANT ROOT FORMATION1*, *LjHAR1*), and soybean (*GmNARK*) display increased lateral root length and density under optimal N growth conditions (Table 1) (Wopereis et al. 2000; Schnabel et al. 2005; Goh et al. 2019; Lagunas et al. 2019). *M. truncatula sunn* mutants continued to produce lateral roots even when tissue N status was high enough to inhibit lateral root growth in the wild type, suggesting a role for MtSUNN in N regulation of root system plasticity (Goh et al. 2019; Lagunas et al. 2019). Interestingly, shoot-to-root auxin transport is elevated in *sunn*, which may explain the promotion of root growth in the mutant (van Noorden et al. 2006; Jin et al. 2012). Therefore, it is hypothesized that MtSUNN regulates resource partitioning between plant organs in a systemic manner (Goh et al. 2019; Lagunas et al. 2019). This hypothesis contrasts with the above-described findings in *Arabidopsis*, where N-dependent lateral root development requires local CLAVATA signaling in the root (Araya et al. 2014). Although, in legumes, a mechanistic link between CLV1-like LRR-RLKs, N-regulated CLE peptides, and root system architecture has not yet been reported, multiple N-induced *CLE* genes were identified in several species (Figs. 1B and 3E) (Okamoto et al. 2009; Lim et al. 2011; Reid et al. 2011; de Bang et al. 2017). In addition, the HPAT mutants *M. truncatula rdn1* and *L. japonicus plenty*, which are defective in CLE arabinosylation, show N-dependent effects on root system architecture (Yoshida et al. 2010; Goh et al. 2019), further supporting a role for an involvement of CLE peptides in root N adaptations across legume species.

In other plant species, N regulation of root plasticity appears to be at least partially independent of the CLAVATA pathway: although multiple tomato (*Solanum lycopersicum*) *CLE* genes (*SlCLE2*, *SlCLE10*, and *SlCLE14*) are upregulated by nitrate (Wulf et al. 2023), recent evidence in tomato and the legume pea (*Pisum sativum*) suggests that the *CLV1*-like orthologs *FASCIATED AND BRANCHED* (*SlFAB*) and *PsNARK* are not involved in lateral root development (Wang et al. 2020). Furthermore, although SlCLV2 and the HPAT FASCIATED INFLORESCENCE (SlFIN) modulate root development, this effect is independent of N availability (Wang et al. 2020).

Together with the data from *Arabidopsis* and *M. truncatula*, these discoveries suggest that the function of the CLAVATA pathway in N-mediated regulation of root system architecture varies among plant species. Although it cannot be excluded that differences in plant N demand or experimental conditions may be partially responsible for this variation, N regulation of root system architecture depends on multiple independent but interconnected local and systemic pathways (Asim et al. 2020). Thus, it is possible that N-responsive pathways are rewired in different plant species, potentially resulting in different relative importance of the pathways on root development. N-mediated root system plasticity is likely particularly complex in legumes, which can engage in nodulation symbiosis with N-fixing rhizobia to satisfy their N needs (discussed below); thus, N and nodulation signaling are tightly connected, and carbon allocation to sustain nodules may compete with carbon allocation to promote root system growth.

### Phosphorus

Phosphorus (P) is essential for major physiological and biochemical processes, with inorganic phosphate (Pi) being the main P source for plants (Paries and Gutjahr 2023). Plants respond to P limitation by various physiological and developmental adaptations, including increases in root-to-shoot ratio and modification of root system architecture to increase Pi uptake (Paz-Ares et al. 2022).

*CLE* genes have been reported to be responsive to Pi starvation or abundance (Fig. 1B). In *Arabidopsis* root tips, several *CLE* genes with Pi starvation-induced expression have been identified, including *AtCLE14* (Gutiérrez-Alanís et al. 2017). *AtCLE14* induction in the root apical meristem of Pi-starving roots requires iron mobilization to these cells, from where the peptide then signals through CLV2 and PEP1 RECEPTOR2 (PEPR2) to maintain meristem identity (Gutiérrez-Alanís et al. 2017). Although not directly shown, it is hypothesized that the AtCLE14-AtCLV2/AtPEPR2 module signals through AtPOL and AtPLL1 to repress the transcription factor genes *AtSHORTROOT* (*AtSHR*) and *AtSCARECROW* (*AtSCR*) (Fig. 3F; Gutiérrez-Alanís et al. 2017; Song et al. 2008), which controls tissue differentiation during root development (Sabatini et al. 2003). Thus, AtCLE14-mediated root meristem termination could explain the primary root growth arrest commonly observed under Pi-limiting conditions (Sanchez-Calderon et al. 2005).

Conversely, expression of other *CLE* genes is induced by high Pi, which may operate as P satiety cues. Such Pi-induced *CLE* genes were identified in several plant species including *L. japonicus* (*LjCLE19* and *LjCLE20*), purple false brome (*Brachypodium distachyon*), and *M. truncatula* (Fig. 1B) (Funayama-Noguchi et al. 2011; Handa et al. 2015; de Bang et al. 2017; Müller et al. 2019; Karlo et al. 2020). *MtCLE33* is induced in roots grown in high Pi conditions; overexpression of *MtCLE33* resulted in MtSUNN-dependent downregulation of strigolactone biosynthesis genes and lower root strigolactone levels relative to controls (Fig. 3F; Müller et al. 2019). Strigolactones are phytohormones known to assist with plant adaptation to Pi starvation by influencing root and shoot plasticity and by modulating plant interaction with microbes (Kohlen et al. 2011; Mayzlish-Gati et al. 2012; Sun et al. 2014). Thus, as discussed below, P-regulated CLE signals may allow plants to integrate plant P status with plant–microbe interactions and may also regulate plant developmental responses to P.

### Sulfur

Sulfur (S) is an essential macronutrient with an important role in the formation of proteins, chlorophyll, and other important compounds (Kopriva et al. 2019). A recent study in *Arabidopsis* provided evidence supporting a regulatory function of CLE peptides in root development in response to S (Fig. 3G) (Dong et al. 2019): long-term limitation of S resulted in a low density of lateral roots in *Arabidopsis* seedlings and gene expression of *AtCLE2* and *AtCLE3* was reduced in seedlings grown under prolonged S deficiency relative to those resupplied with S (Fig. 1B). As noted above, these genes have described roles in lateral root development and carbon allocation in response to other abiotic cues including light/dark and N (Fig. 3, B and E). Interestingly, under prolonged S deficiency, *clv1* mutants displayed a higher daily increase rate of lateral root density relative to wild-type plants (Dong et al. 2019). Although the precise mechanisms remain to be fully elucidated, *AtCLE2* and *AtCLE3* expression increased in *clv1* mutants compared to the wild type under S deficiency, indicating the existence of a negative, CLV1-dependent feedback mechanism that regulates *CLE* gene expression and lateral root development (Fig. 3G) (Dong et al. 2019). S-responsive *CLE* genes were also identified in *M. truncatula* (Fig. 1B) (de Bang et al. 2017). Although not functionally investigated, this finding indicates that other plant species may possess mechanisms similar to *Arabidopsis* to adapt root system plasticity in response to S.

### Other macronutrients and micronutrients

Our knowledge on nutrient-responsive *CLE* genes remains limited to the macronutrients N, P, and S. Transcriptional profiling of signaling peptides revealed 18 *M. truncatula CLE* genes responsive to N, P, and/or S in roots or shoots; however, intriguingly no K-regulated *CLE* genes were reported (de Bang et al. 2017). While it could be that *CLE* signaling is specific to certain nutrients and does not operate in K signaling, it is also possible that the lack of K-regulated *CLE* genes is specific to *M. truncatula* or the growth conditions used in the study (de Bang et al. 2017). To our knowledge, *CLE* expression in response to other nutrients was not tested in any plant species. Nevertheless, it is conceivable that CLAVATA signaling also regulates plant responses to other macronutrients or micronutrients as there is extensive crosstalk between different nutrient signaling and uptake pathways (Fan et al. 2021). For example, P and iron (Fe) uptake shares an inverse relationship as Fe deficiency promotes P uptake and P deficiency increases Fe availability in plants (Xie et al. 2019). Numerous *CLE* genes are responsive to P, and, as noted above, Fe mobilization in the root apical meristem is required to induce *AtCLE14* expression in response to low Pi availability (Gutiérrez-Alanís et al. 2017). Thus, investigating whether P deficiency and Fe availability induce a similar set of *CLE* genes will offer valuable insights into nutrient crosstalk and the associated signaling pathways. In any natural environment, plants are simultaneously exposed to a multitude of stimuli, including information about the availability of various nutrients. Plant developmental adaptations such as root system plasticity respond to a plethora of cues, including combinations of nutrient deficiencies (Bouain et al. 2019). If or how combinations of nutrient-responsive CLE signals are integrated during plant nutrient homeostasis should be further studied.

## CLAVATA signaling integrates nutrient and symbiosis signaling during plant–microbe interactions

Plant CLE peptides and CLAVATA-type receptors have been extensively studied in the context of symbiosis regulation. Below, we review the roles of CLAVATA signaling during nodulation and arbuscular mycorrhiza (AM) symbiosis and discuss how interconnected CLE pathways allow plants to integrate symbiosis and nutrient status. In contrast to the above-described CLE peptides coordinating plant developmental adaptations to abiotic stimuli, many CLE peptides involved in plant–biotic interactions elicit no apparent developmental changes in the plant, indicating that CLE-mediated nutrient and symbiosis homeostasis may be uncoupled from developmental adaptations. However, because CLAVATA1 receptors regulate nutrient homeostasis, symbioses, and plant development, these processes are at least partially interconnected (see below).

### CLE peptides mediate AON and integration with plant N and P status

Legumes engage in nodulation symbiosis with N-fixing bacteria (rhizobia), which are harbored in specialized root organs (nodules) and provide their host with N in exchange for carbon (Roy et al. 2020). To prevent excessive nodule formation, legumes have evolved a sophisticated regulatory mechanism called autoregulation of nodulation (AON) (Chaulagain and Frugoli 2021). AON is a complex, systemic signaling system modulated by a root-derived, nodulation-induced CLE peptide signal, which travels to the shoot. CLE perception by shoot-acting receptors then initiates a shoot-to-root signal that suppresses further nodulation. A similar system operates during N control of nodulation. AON and N control of nodulation have been extensively studied in the legumes *L. japonicus*, *M. truncatula*, common bean (*Phaseolus vulgaris*), soybean, and pea. Here, we will focus only on the contribution of CLAVATA signaling to these pathways; for more in-depth information on AON, please refer to recent reviews (Chaulagain and Frugoli 2021; Li et al. 2022).

Expression of *L. japonicus CLE-ROOT SIGNAL1* (*LjCLE-RS1*), *LjCLE-RS2*, *LjCLE-RS3*, *LjCLE40*, *LjCLE3*, and *LjCLE16*; *M*. *truncatula CLE12*, *MtCLE13*, *MtCLE34*, and *MtCLE35*; *G. max RHIZOBIA-INDUCED CLE1* (*GmRIC1*) and *GmRIC2*; *P. vulgaris RIC1* and *PvRIC2*; and *P. sativum PsCLE12*, *PsCLE13*, *PsCLE12-like*, and *PsNIC* is induced in roots inoculated with rhizobia (Okamoto et al. 2009; Mortier et al. 2010; Lim et al. 2011; Reid et al. 2011; Ferguson et al. 2014; Nishida et al. 2016; Kassaw et al. 2017; Samorodova et al. 2018; Isidra-Arellano et al. 2020; Yoro et al. 2020; Mens et al. 2021). Overexpression of these genes inhibits nodulation, indicating that they act as negative regulators of legume–rhizobia symbiosis (Okamoto et al. 2009; Mortier et al. 2010; Lim et al. 2011; Reid et al. 2011; Okamoto et al. 2013; Ferguson et al. 2014; Nishida et al. 2016; Isidra-Arellano et al. 2020). Posttranslational arabinosylation of CLE peptides is required for functional AON: MtCLE12 (but not MtCLE13) requires the root-acting HPAT MtRDN1 to repress nodule formation (Kassaw et al. 2017), and LjCLE-RS1/2/3 are fully or partially dependent on the HPAT LjPLENTY (Yoro et al. 2020), suggesting both HPATs act in a substate-specific or substate-preferential manner.

CLE-mediated suppression of nodulation depends on shoot-acting CLV1-like LRR-RLKs (LjHAR1/MtSUNN/GmNARK/PsSYMBIOSIS 29 [PsSYM29]/PvNARK) (Table 1) (Krusell et al. 2002; Okamoto et al. 2009; Mortier et al. 2010; Lim et al. 2011; Ferguson et al. 2014). This suggests that nodulation-induced CLE peptides travel through the vascular tissue from the roots to the shoots. Legume *clv1* mutants exhibit a hypernodulation phenotype characterized by excess numbers of nodules, indicating that CLV1 receptors are negative regulators of symbiosis (Ferguson et al. 2014; Okamoto and Kawaguchi 2015; Crook et al. 2016; Nowak et al. 2019). Additional proteins are involved in CLE perception during AON: the LRR-RLKs LjHAR1 and LjKLAVIER form a receptor complex responsible for transmitting LjCLE-RS1 and LjCLE-RS2 signals (Miyazawa et al. 2010; Krusell et al. 2011). In addition, *L. japonicus* and pea *clv2* (*sym28*) mutants are hypernodulating (Krusell et al. 2011); mutant studies suggest that LjHAR1 and LjCLV2 act in the same AON pathway as nodule numbers in *har1 clv2* double mutants do not differ from those in *har1* (Krusell et al. 2011). Furthermore, the *M. truncatula* pseudokinase MtCRN has been found to be a shoot-acting component of MtCLE12 and MtCLE13 signaling (Crook et al. 2016; Nowak et al. 2019). In *Arabidopsis*, the receptor-like protein AtCLV2 and AtCRN are commonly considered to form a CLE perception unit (Box 1; Fig. 2), and although, during nodulation, the 2 genes have not yet been studied in the same plant species, it is likely that they act in concert also in the context of AON. Indeed, heterologous expression of MtCLV2 and MtCRN in *Nicotiana benthamiana* leaf epidermal cells revealed that these 2 plasma membrane proteins can form heteromers with each other, as well as with MtSUNN (Crook et al. 2016). Conversely, similar colocalization studies of LjHAR1 and LjCLV2 did not show heteromer formation (Krusell et al. 2011), which could be due to the instability of the complex without CRN (Fig. 2) (Bleckmann et al. 2010). Overall, our knowledge on CLE-receptor complex(es) in AON is fragmented across legume species and requires further study. Because *L. japonicus har1 plenty* double mutants display an additive nodulation phenotype (Yoro et al. 2020), the existence of at least 2 partially independent CLE signaling pathways regulating nodule number should be considered and carefully investigated.

Perception of CLE peptides by shoot-acting receptors eventually triggers a shoot-to-root downstream signal. Cytokinin was proposed to be the downstream signal originating from the shoot (Sasaki et al. 2014). Furthermore, AON signal perception by LjHAR1 results in the repression of the micro-RNA *miR2111* in the shoot; miR2111 accumulates in low N conditions and is translocated to the root, where it creates susceptible conditions for rhizobia infection by repressing the F-box protein *TOO MUCH LOVE(TML)* (Fig. 3H; Table 1) (Tsikou et al. 2018; Gautrat et al. 2020; Okuma et al. 2020).

N availability is one of the main regulators of nodulation symbiosis as nodulation is suppressed in plants grown under sufficient N (Imsande 1986). Nodulation-induced *LjCLE-RS2*, *LjCLE-RS3*, *MtCLE34*, *MtCLE35*, *LjCLE40*, *PsCLE12*, *PsCLE13*, and *NITRATE-INDUCED CLE* (*PsNIC*) are also induced by N (Okamoto et al. 2009; Nishida et al. 2016; Moreau et al. 2021; Lebedeva, Dvornikova, and Lutova 2022; Lebedeva, Sadikova, et al. 2022). Conversely, *GmNIC1*, *GmNIC2*, and *PvNIC1* are induced in roots grown under high N conditions but not in response to rhizobia (Reid et al. 2011; Ferguson et al. 2014). With the notable exception of *MtCLE34*, which is considered a pseudogene due to a premature stop codon (Mens et al. 2021), overexpression of N-induced soybean, *L. japonicus*, and *M. truncatula CLE* genes represses nodulation (Okamoto et al. 2009; Reid et al. 2011, 2013; Ferguson et al. 2014; Nishida et al. 2016; Moreau et al. 2021; Lebedeva, Dvornikova, and Lutova 2022). This suggests that these genes play an essential role in integrating plant N needs with symbiosis status. Recent research revealed intriguing mechanistic details on the intersection of AON and N-dependent regulation of nodulation: *MtCLE35* induction by high N requires the transcription factor *MtNLP1*, which has been reported as a key regulator involved in the N-mediated suppression of nodulation in *M. truncatula* (Lin et al. 2018; Nishida et al. 2018; Lebedeva et al. 2020; Luo et al. 2021; Moreau et al. 2021). Like AON-associated CLE peptides, MtCLE35 also acts through root-acting MtRDN1, shoot-acting MtSUNN, and the miR2111 systemic effector to inhibit nodulation (Fig. 3E), suggesting that reducing the expression of *MtCLE35* or increasing *miR2111* accumulation can bypass the inhibitory effects of N on nodulation (Mens et al. 2021; Moreau et al. 2021). Understanding the mechanisms underlying the complex interaction of CLE peptides, MtSUNN, and miR2111 has substantial implications for crop development with improved N use efficiency.

In addition to N availability, other abiotic cues also regulate nodulation. For example, nodulation is suppressed in plants grown under P-limiting conditions (Tang et al. 2001). Interestingly, the *P. vulgaris CLE* genes *PvRIC1* and *PvRIC2* are upregulated in roots not only when inoculated with rhizobia but also when grown under low P conditions (Isidra-Arellano et al. 2020), revealing a high degree of complexity in the interaction between CLE peptides and external environmental stimuli. In addition, studies in pea, soybean, and common bean revealed that P-mediated control of nodule number depends on *PsNARK/GmNARK/PvNARK* (Foo et al. 2013; Isidra-Arellano et al. 2020). Thus, AON and N and P regulation of nodulation require the same CLV1-tye LRR-RLKs, suggesting that these receptors integrate various CLE signals to allow the plant to balance nutrient needs, symbiosis, and developmental adaptations (see below). Notably, nodulation is also influenced by abiotic stresses such as drought (Lumactud et al. 2023), which is known to induce certain *CLE* genes in other plant systems (see above); however, it remains to be determined if drought regulation of nodulation requires CLAVATA signaling.

### CLE-mediated AM symbiosis autoregulation and crosstalk with plant N and P status

AM symbiosis is a ubiquitous association between plants and Glomeromycotina soil fungi. Among other benefits, AM fungi provide the plant with essential nutrients, most notably P but also others including N, in exchange for photosynthetic products (Keymer and Gutjahr 2018). Like nodulation symbiosis, the extent of AM symbiosis is controlled by plant nutrient status—with P being the most-studied one—as well as by an autoregulatory signaling pathway (autoregulation of mycorrhizal symbiosis [AOM]) (Müller and Harrison 2019). AOM involves signaling between the roots and shoots of the plant and is thought to be critical for maintaining the balance between the benefits of AM fungal root colonization and the symbiotic carbon costs (Vierheilig et al. 2000).

Transcriptomic analyses revealed upregulation of several *CLE* genes in AM fungus-colonized roots of *M. truncatula*, *B. distachyon*, tomato (Fig. 1B), and *L. japonicus* (*LjCLE7*, *LjCLE15*, *LjCLE19*, *LjCLE20*, *LjCLE24*, and *LjCLE29*) (Handa et al. 2015; Müller et al. 2019; Karlo et al. 2020; Wulf et al. 2023). Further investigation revealed that *MtCLE53* is expressed in the stele of roots colonized by AM fungi (Müller et al. 2019; Karlo et al. 2020). Ectopic overexpression of *MtCLE53* in roots of *M. truncatula* resulted in decreased fungal root colonization, achieved by modulating the synthesis of symbiosis-promoting strigolactones (Fig. 3I) (Müller et al. 2019). Conversely, colonization levels were higher in *cle53* mutants than in wild-type plants (Karlo et al. 2020). These findings suggest that AM-induced *MtCLE53* is a negative regulator of AM fungal colonization. Like AON, RDN1-mediated arabinosylation of CLE peptides is necessary for AOM: HPAT mutants in *M. truncatula* (*rdn1*) and tomato (*fin*) displayed elevated number of arbuscules and vesicles relative to wild-type plants (Karlo et al. 2020; Wang, Veladia, et al. 2021). Furthermore, *M. truncatula rdn1* was insensitive to *MtCLE53* overexpression (Karlo et al. 2020), confirming the importance of HPAT-mediated CLE arabinosylation for AOM.

Perception of AOM-associated CLE peptides requires a receptor complex similar to AON: mutants in the *CLV1*-orthologs of legumes (*MtSUNN*, *LjHAR*, *PsSYM29*, and *GmNARK*) and nonlegumes (*BdFON1* and *SlFAB*), as well as the tomato *clv2* mutant, display hypercolonization phenotypes characterized by elevated overall AM fungal root colonization and arbuscule numbers (Morandi et al. 2000; Solaiman et al. 2000; Meixner et al. 2005; Müller et al. 2019; Wang et al. 2018; Wang, Velandia, et al. 2021). While direct binding has not been shown, MtCLE53 function depends on MtSUNN, indicating the 2 proteins act in the same pathway (Müller et al. 2019; Karlo et al. 2020). Interestingly, AM-induced SlCLE11, which is closely related to MtCLE53, also suppresses AM symbiosis but acts independently of SlFAB or SlCLV2, suggesting the existence of multiple distinct CLE-mediated signaling modules involved in symbiosis regulation (Wulf et al. 2023). Although AOM was described in *Hordeum vulgare* (barley) as a systemic process (Vierheilig et al. 2000) and although AOM and AON depend on an overlapping set of LRR-RLKs, it is unclear if the perception of root-derived AOM-associated CLE peptides by CLV1 receptors also occurs in the shoot: while split-root experiments in soybean suggested that *GmNARK* acts in a systemic manner to modulate AM colonization, grafting experiments in tomato indicated that *SlFAB* acts locally in the roots and not the shoots to suppress AM symbiosis (Meixner et al. 2005; Schaarschmidt et al. 2013; Wang, Velandia, et al. 2021). Furthermore, SlCLV2 acts in both the root and the shoot (Wang, Velandia, et al. 2021), suggesting the existence of systemic and local CLAVATA-mediated signaling pathways that control AM fungal root colonization. Despite the striking similarities of AM and nodulation autoregulation and although either symbiosis can suppress the development of the other (Catford et al. 2003), AOM and AON display marked specificity: nodulation-induced *MtCLE13* is not induced by AM symbiosis and *MtCLE13* overexpression does not impact AM symbiosis, suggesting that symbiotic CLE peptides have distinct roles (Müller et al. 2019).

Like nodulation symbiosis, AM symbiosis is not only autoregulated but also controlled by plant nutrient status: P starvation induces the production of symbiosis-promoting strigolactones, and high plant P status systemically suppresses AM symbiosis (Müller and Harrison 2019). Interestingly, some of the AM-induced *CLE* genes in *B. distachyon*, *M. truncatula*, and *L. japonicus* (*LjCLE19* and *LjCLE20*) are also regulated by P (Fig. 1) (Funayama-Noguchi et al. 2011; Handa et al. 2015; Müller et al. 2019; Karlo et al. 2020). Overexpression of the P-induced *MtCLE33* in transgenic *M. truncatula* roots resulted in a decrease in AM root colonization and negatively regulated strigolactone biosynthesis (Müller et al. 2019), suggesting that P-induced *MtCLE33* and the AOM signal *MtCLE53* act in concert to fine-tune AM fungal root colonization by modulating strigolactone content in response to P status and symbiosis, respectively (Fig. 3, F and I). The effect of other P-induced *CLE* genes on AM symbiosis remains unknown, but exploring these could shed light on the intricate balance of P nutrition and AM symbiosis. Importantly, P suppression of AM symbiosis is at least partially independent of CLV1, as *clv1* mutants in *M. truncatula*, soybean, and pea still displayed reduced AM fungal root colonization when grown in high P conditions (Wyss et al. 1990; Foo et al. 2013; Müller et al. 2019). Intriguingly, neither *sunn* nor mutants of the pea and tomato orthologs *PsNARK* and *SlFAB* show increased strigolactone content or elevated expression of strigolactone biosynthesis genes (Foo et al. 2014; Wang et al. 2020). However, mutants in the *P. sativum* HPAT *rdn1* displayed significantly elevated levels of strigolactones (Foo et al. 2014). It is therefore conceivable that RDN1-arabinosylated CLE peptides fine-tune strigolactone biosynthesis independent of CLV1-like LRR-RLKs or that a compensatory pathway exists that modulates CLE-regulated strigolactone biosynthesis in the absence of *CLV1*.

AM symbiosis is also regulated by plant N status (Liu et al. 2012). CLV1-type receptors appear to play an important role in plant adaptation to N availability (see above). In addition, tomato *SlFAB* and *SlFIN* were required for N (but not P) suppression of AM (Wang, Velandia, et al. 2021). In addition, a recent study in tomato identified SlCLE10 as a N- and AM-induced CLE peptide, although its effect on AM fungal colonization appears minor (Wulf et al. 2023).

Taken together, evidence from different plant species suggests that components of the CLAVATA pathway regulate AM autoregulation as well as integration with plant P and N status. Although CLAVATA signaling in the context of AM symbiosis is less understood than during nodulation symbiosis, it becomes evident that nutrient and autoregulation pathways of both symbioses share similarities but also striking differences; some of these may depend on the plant species or experimental conditions. As described above, many *CLE* genes are induced by abiotic factors and AM symbiosis aids in plant resistance against abiotic stresses such as drought, salinity, and cold (Augé et al. 2015; Li et al. 2019; Pedranzani et al. 2016; Wang, Dhroso, et al. 2021; Cosme 2023). Thus, it is conceivable that AM symbiosis may be regulated by additional CLE peptides responsive to such abiotic cues. Careful comparative studies with multiple plant species and environmental conditions are required to shed light on the complex interaction of multiple, potentially overlapping CLE pathways governing host interactions with AM fungi.

### CLAVATA receptors modulate plant–pathogen interactions

Apart from symbiotic interactions with beneficial microbes such as rhizobia and AM fungi, the CLAVATA pathway is also implicated in plant interactions with pathogenic bacteria, oomycetes, and fungi (Hanemian et al. 2016). *Arabidopsis clv1*, *clv2*, and *crn* mutants were more resistant to infections with the bacterial wilt pathogen *Ralstonia solanacearum* and the oomycete *Hyaloperonospora arabidopsidis* than wild-type plants. Conversely, the mutants were more susceptible to infections by the bacterial pathogen *Pseudomonas syringae* and 2 species of necrotrophic fungi including *Botrytis cinerea*. Interestingly, the effects on plant–pathogen interactions seem specific to AtCLV1, AtCLV2, and AtCRN; neither known other players of the meristematic CLAVATA signaling pathways (AtCLV3 and AtBAM) nor downstream signaling components (AtPOL and AtWUS) are required (Hanemian et al. 2016). Instead, resistance to *R. solanacearum* in *clv1* mutants corresponds to accumulation of the microRNA *mi169* and NUCLEAR FACTOR Y subunit alpha (NF-YA) transcription factors, indicating that CLAVATA signaling during plant–pathogen interactions strikingly differs from that during shoot meristem maintenance (Fig. 3J) (Hanemian et al. 2016). It is currently unknown which CLE peptides act upstream of CLV1, CLV2, and CRN in the context of plant–pathogen interactions. In the quest for their identification, researchers should consider that these could be either plant-derived or produced by the microbes (see below).

## The CLAVATA pathway mediates cross-kingdom signaling with plant-interacting organisms

CLE peptides were long thought to be specific to plants; however, accumulating evidence indicates that some plant-interacting organisms encode CLE-like peptides. Plant CLAVATA-type receptors interact with CLE-like signaling peptides from plant-interacting organisms, which hijack the plant CLAVATA signaling pathway for their own benefit.

### Parasitic nematodes secrete CLE-like effectors in planta

Parasitic cyst nematodes feed on their host plants by establishing syncytial feeding sites in roots. Upon infection, nematodes select a single cell near the root vasculature, typically a procambial or pericycle cell, to initiate syncytium formation (Davis and Mitchum 2005). The nematode uses its stylet to deliver effector proteins, which mimic host peptides and allow the parasite to evade the host's immune system (Wang et al. 2005; Frei dit Frey and Favery 2021). Among these are CLE-like peptides, which nematodes secrete into the root cell to stimulate feeding cell formation (Davis et al. 2008; Gardner et al. 2015). Secretion of CLE propeptides from the nematode esophageal glands into plant cells is a notable example of molecular mimicry (Olsen and Skriver 2003; Wang et al. 2005). Nematode CLE propeptides hijack the host cells secretory pathway and are processed by the host cells; subsequent perception of nematode-derived mature CLE-like peptides by host CLAVATA receptors results in developmental reprogramming of root tissues (Wang et al. 2010; Guo et al. 2011; Wang et al. 2011; Wang, Dhroso, et al. 2021). For example, HsCLE1 and HsCLE2 from the beet cyst nematode *Heterodera schachtii* exhibit striking similarities to AtCLE1 to 7; of particular interest was the discovery that the active peptide encoded by *HsCLE2* is identical in sequence to that of AtCLE5 and AtCLE6 (Wang et al. 2011). When ectopically applied, HsCLE2 affected root and shoot development like the *Arabidopsis* peptides (Wang et al. 2010). HsCLE1 and HsCLE2 peptides are perceived by host receptors including AtCLV1, the AtCLV2/AtCRN heterodimer and AtRPK2, and their orthologs in soybean (Fig. 3K; Table 1) (Replogle et al. 2011; Replogle et al. 2013; Guo et al. 2015). Additionally, a family of CLE-like peptides from cyst nematodes resembling *Arabidopsis* TDIF (HsCLEB) also play a role in parasitism by signaling through the hosts the AtTDIF-AtTDR (TDIF receptor)-WUSCHEL-RELATED HOMEOBOX4 (WOX4) pathway (Guo et al. 2017). Furthermore, GrCLE1, which is produced by the potato cyst nematode *Globodera rostochiensis*, is processed by plant proteases after injection and binds directly to AtBAM1, AtBAM2, and AtCLV2 (Fig. 3K) (Guo et al. 2011).

Not only nematode-derived but also plant-derived CLE peptides play a role in plant–nematode interactions: recently, it was demonstrated that the expression of *Arabidopsis* genes *AtCLE1*, *AtCLE3*, *AtCLE4*, and *AtCLE7* is significantly induced in galls that develop on roots infected by the root-knot nematode *Meloidogyne incognita* (Nakagami et al. 2023). Through loss-of-function analyses, it was observed that *cle3* and *clv1* mutants displayed fewer galls, while estradiol-induced overexpression of *AtCLE3* resulted in an increased number of galls. AtCLE3 interacts with the AtCLV1 receptor, suggesting that a plant CLE-CLV1 module is required to facilitate gall formation (Fig. 3K) (Nakagami et al. 2023). Similar results were obtained in *M. truncatula,* where mutants in the *CLV1* ortholog *MtSUNN* displayed reduced gall numbers when infected with the root-knot nematode *Meloidogyne javanica*; however, this effect seemed to depend on the density of nematodes in the inoculum (Costa et al. 2020). A better understanding of the mechanisms underpinning the interplay of plant and nematode CLE peptides, including their perception by plant LRR-RLKs, may help to develop new tools for crop protection. Furthermore, genome mining for *CLE*-like genes in other plant parasites should be performed to determine if other plant-interacting organisms employ similar infection strategies targeting the host CLAVATA pathways.

### Mutualistic AM fungi express CLE-like genes in planta

So far, the only other known groups of plant-interacting organisms producing CLE-like peptides are mutualistic AM fungi: *CLE*-like genes were found in 4 *Rhizophagus* species and 1 *Gigaspora* species; however, no *CLE*-like genes could be identified in other sequenced AM fungal genomes (Le Marquer et al. 2019). Transcript analysis of *Rhizophagus irregularis CLE1* (*RiCLE1*) and *Gigaspora rosea CLE1* (*GrCLE1*) revealed that both genes are strongly induced in planta (symbiotic condition) relative to germinating spores and extraradical mycelium (asymbiotic condition). Synthetic RiCLE1 peptide application promotes AM fungal colonization by stimulating both the entry and the spread of the fungus in roots (Le Marquer et al. 2019). Moreover, RiCLE1 peptide treatment caused primary root growth inhibition in *Arabidopsis*, *M. truncatula*, and pea: an effect that was partially dependent on AtCLV2 but not on AtCLV1 (Fig. 3L). Because AM fungal CLE peptides show high sequence similarity with those of plants, this evidence suggests that they may be perceived by host CLE receptors. In addition, RiCLE1 peptide treatment stimulated lateral root formation in *M. truncatula* (Le Marquer et al. 2019). Because AM fungi modulate root system architecture and preferentially colonize lateral roots (Gutjahr and Paszkowski 2013; Chiu et al. 2022), it is tempting to speculate that AM fungus-derived CLE-like peptides may act as effectors promoting fungal root colonization by mimicking plant responses to low P. However, more research is required to better understand if and how fungal CLE-like peptides are perceived by the host plant and with which endogenous host CLAVATA pathways RiCLE1 interferes (if any).

## Integration of environmental and developmental signals by CLAVATA signaling

**Figure 4.**
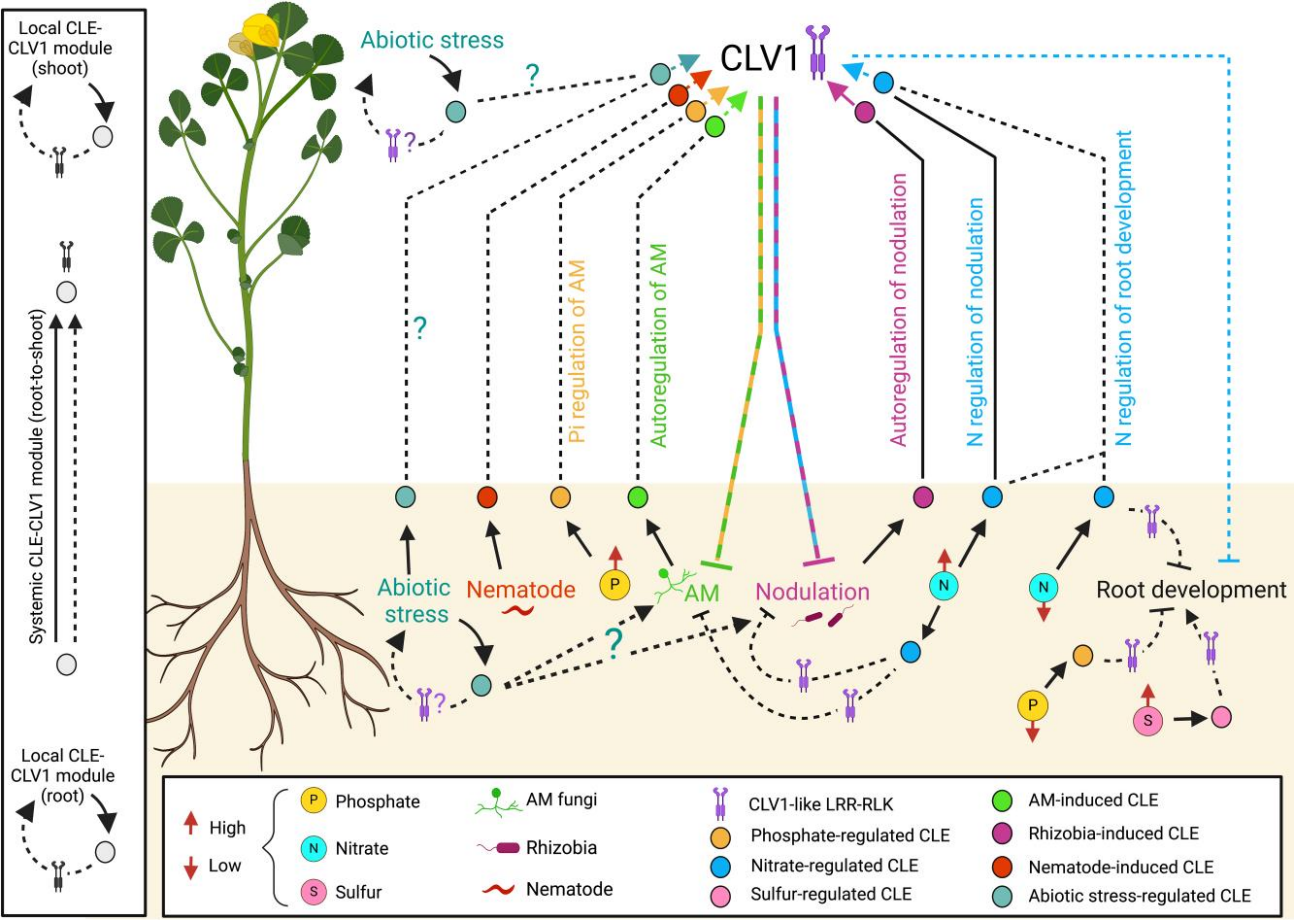
Model for CLAVATA1 as a central hub integrating multiple CLE signaling pathways. CLE-CLV1 modules act as a central component for the integration of signals related to abiotic stress, nutrient availability, AM symbiosis, nodulation, plant–pathogen interactions, and development. Root-derived CLE signals can be perceived by CLV1 receptors localized in the shoot (systemic) or root (local). Other known CLE signaling modules act locally in the shoot. Solid lines depict established connections, while dashed lines indicate putative/potential or indirect associations. Arrows represent activating signals, and blunt ends indicate repressive signals. P and N influence AM symbiosis and nodulation via partially overlapping signaling pathways. Both symbioses are also fine-tuned by partially overlapping autoregulation pathways. Shared inhibitory signals between autoregulation and nutrient signaling are represented by overlapping dashed lines in different colors. Question marks (?) symbolize research gaps that represent potential links in the pathway yet to be investigated, including the role of abiotic stress-induced CLE signals on symbioses. Environmental cues are denoted by symbols explained at the bottom of the figure. Red arrows on nutrient symbols (P, S, and N) indicate their levels (high or low). Figure was created with BioRender.com.

The number of studied CLAVATA-type receptors in vascular plants is relatively low compared to the large number of reported CLE peptides in plant genomes (Fletcher 2020). For example, *Arabidopsis* encodes 33 *CLE* genes but only a handful of bona fide CLE-binding receptors (e.g. *AtCLV1*, *AtBAM1/2/3*, and *AtTDR*) (Carbonnel et al. 2023). The LRR-RLK CLV1 is particularly well studied and known to play diverse roles in plant development, physiology, and plant–environment interactions by interacting with a variety of CLE peptides (Fig. 4; Table 1).

### CLV1 serves as hub for multiple CLE signaling pathways

An essential strategy to respond and adapt to environmental changes is phenotypic plasticity, where a plant can alter its physiology or morphology. Plant plasticity is associated with developmental changes and carbon partitioning; both are strongly influenced by the availability of nutrients, pathogens, abiotic stresses, and the microbiome in the rhizosphere (Goh et al. 2013). CLV1 LRR-RLKs, being a putative hub for signal integration of diverse environmental cues, may play an essential role in phenotypic plasticity by interacting with various CLE peptides (Fig. 4). Local and systemic CLE-CLV1 signaling modules influence N-, P-, and S-dependent root development. Shoot-active legume CLV1 orthologs play an important role in AON by perceiving nodule-induced, root-derived CLE peptides; in legumes and nonlegumes, CLV1 receptors act as regulators of AOM by perceiving AM-induced CLE peptides (Fig. 4; Table 1) (Wang et al. 2018). Both autoregulatory processes have been suggested to prevent oversequestration of carbon by the microbial symbiont (Wang et al. 2018). In line with this, accumulating evidence suggests that systemic, CLV1-dependent signaling pathways are associated with carbon partitioning toward the roots also in other contexts: root galls formed by parasitic root-knot nematodes constitute strong carbon sinks (Carneiro et al. 1999), and their development is regulated by a long-distance *AtCLE3-AtCLV1*-dependent pathway (Nakagami et al. 2023). Furthermore, AtCLE2 and AtCLE3 are proposed to act as systemic root-to-shoot signals integrating root carbon availability with root system architecture, potentially also in concert with AtCLV1 (Araya et al. 2014; Ma et al. 2020; Okamoto et al. 2022; Nakagami et al. 2023). Because *AtCLE1* to *7* and closely related peptides of other plant species are induced in roots by a variety of environmental stimuli (Fig. 1B), it is tempting to speculate that these peptides may be part of a universal, CLV1-dependent signaling module regulating carbon allocation and root responses to a variety of environmental signals.

### Signaling specificity at the CLE-receptor module

Mature CLE peptides have highly conserved sequences (Fig. 1A) and their diverse functions depend on a relatively small group of LRR-RLKs. Although signaling specificity of closely related CLE peptides has been reported in some cases (Müller et al. 2019), it is unknown how CLE-receptor complexes distinguish between different CLE peptides to elicit a specific cellular response. Tight spatiotemporal expression control of *CLE* genes and their receptors, dosage-dependent competition among CLE peptides for the same receptor, unique posttranslational modifications of CLE peptides, and/or CLE-specific binding affinities to different receptor complexes may be some of the ways how specific signaling can be achieved (Roy and Müller 2022). In *Arabidopsis*, tight spatiotemporal regulation of peptide and receptor expression is critical for specific CLE function, and 2 antagonistic CLE-receptor modules were reported to operate in the central zone (AtCLV3-AtCLV1) and peripheral zone (AtCLE40-AtBAM1) of the shoot apical meristem (Schlegel et al. 2021). Conversely, several environment-responsive CLAVATA signaling pathways are proposed to act on a systemic level involving root-derived CLE peptides and shoot-acting CLV1 receptors (Fig. 4). In such cases, multiple CLE peptides may be found in the xylem sap at the same time, where they may compete for the same receptor(s). Receptors distinguish between various ligands based on their amino acid sequence and/or posttranslational modifications. In vitro structure and binding studies with AtBAM1-AtCLE9 (CLE Cluster 3B, Fig. 1B) and AtTDR-AtTDIF (AtCLE41/44, Cluster 4, Fig. 1B) revealed that the first 3 amino acids of the N-terminus of the CLE peptides are required to specifically bind to their cognate receptor (Zhang et al. 2016; Li et al. 2017; Roman et al. 2022). For AtTDIF, an additional C-terminal anchoring site was described (Zhang et al. 2016; Li et al. 2017). However, it is unclear if or how receptors distinguish between CLE peptides that share the N-terminal 3 amino acids but contain variable residues in Positions 4 to 12 (Fig. 1A).

### Signaling pathways downstream of CLE perception

Our knowledge on signaling downstream of the CLE-CLV1 module is fragmented (Fig. 3). However, even the limited data that are available suggest that signaling downstream of CLE perception is at least partially context specific: while CLV1 and CLV2 in the context of shoot meristem maintenance regulate the transcription factor WUSCHEL (Box 1; Fig. 2) (Somssich et al. 2016), CLV1-dependent downstream signaling in response to interaction with *R. solanacearum* is WUSCHEL independent and instead involves the microRNA miR169 and NF-YA transcriptional regulators (Hanemian et al. 2016). Interestingly, NF-YA proteins are also implicated in *Gm*NARK-dependent autoregulation of AM symbiosis in soybean (Schaarschmidt et al. 2013) and are required for nodule development (Laloum et al. 2014), indicating that this transcriptional regulator may act downstream of CLV1 during diverse plant–biotic interactions. Although a direct link to CLE peptides remains elusive, recent data indicate that signaling downstream of CLV1 differs even between closely related plant–microbe symbioses as the RNA Polymerase II mediator subunit *MED16a*, which was identified in a suppressor screen of the *sunn* hypernodulation phenotype, acts as a positive regulator of nodule number and root elongation but negatively impacts AM symbiosis (Chaulagain et al. 2023). RNA Polymerase II mediators impact gene expression by forming a bridge between transcriptional activators at the enhancer region and the transcriptional machinery (Kyrchanova and Georgiev 2021). Thus, uncovering the transcriptional regulons impacted by MED16a may allow the identification of specific signaling components downstream of CLV1 during symbiosis, root development, and potentially other contexts.

## Concluding remarks: challenges and opportunities for future research

Although many *CLE* genes are reported to be differentially regulated in response to environmental stimuli, for the majority, an experimental validation of their function is lacking (Fig. 1B). One reason for this discrepancy is that genetic studies of CLE signaling are challenging (Fletcher 2020). Due to the small size of *CLE* genes, relatively few transposon insertion mutants are readily available. Thus, functional studies with *cle* mutants have only become more feasible with the increasing availability of CRISPR/Cas9-mediated targeted genome editing in plants (Yamaguchi et al. 2017; Rodriguez-Leal et al. 2019; Liu et al. 2021). But even if mutants are available, researchers face the problem that CLE peptides often act redundantly, which makes pinpointing a phenotype to a specific *CLE* gene challenging (Song et al. 2021). In addition, CLAVATA pathways are characterized by substantial functional redundancy and the loss of a CLE peptide or CLAVATA-type receptor can often be compensated by closely related proteins (Rodriguez-Leal et al. 2019; Kwon et al. 2022). To circumvent some of the issues associated with mutant studies, many researchers turned to overexpression and/or synthetic peptide treatment experiments (e.g. Mortier et al. 2010; Endo et al. 2013; Le Marquer et al. 2019). While such experiments have greatly advanced our understanding of CLE function, they should also be treated with some caution. Due to redundancy and compensation mechanisms of CLAVATA pathways, overexpression or local application of a CLE to cell types that it is not normally expressed in may result in nonspecific phenotypes. It is also conceivable that any ectopically applied CLE peptide taken up by the roots can enter the xylem stream and elicit a phenotype in the shoot, which may lead researchers to conclude they act as systemic signals. To avoid such issues, suitable controls such as scrambled peptides should be employed when conducting peptide treatment experiments and *CLE* overexpression should be ideally performed in a cell-type–specific manner.

However, the spatiotemporal expression pattern of most environment-regulated *CLE* genes remains elusive (see Outstanding Questions section). Studies that reported *CLE* expression level changes in response to environmental cues can be difficult to interpret as they often result from bulk transcriptome sequencing of whole seedlings, roots, or shoots (Fig. 1B). Therefore, high-resolution spatiotemporal expression analyses for environment-regulated *CLE* genes are urgently needed. Recent technological advances including single-cell/single-nucleus and spatial transcriptomics are being increasingly adopted for plant tissues (Liu et al. 2023; Nobori et al. 2023; Yu et al. 2023), and we anticipate that such approaches will greatly advance our understanding of complex CLE signaling networks under various environmental conditions. Similarly, there is a need for a thorough investigation of the regulatory networks upstream of environment-responsive *CLE* genes, including identifying the transcriptional regulators governing their expression. Such investigations are important to better understand why a single *CLE* can respond to multiple stimuli and how the regulation of *CLE* transcription by a single stimulus is coordinated (Fig. 1B).

Moreover, the processes at the CLE-receptor interface remain largely elusive. Although components of CLE-receptor complexes, such as LRR-RLKs, coreceptors, and kinases, have been identified using mutant studies and in vitro binding assays (Fig. 2), it is still largely unclear which receptor complexes exist in vivo and in which cell type. We expect that sophisticated high-resolution omic approaches, including single-cell proteomics (Clark et al. 2022), will advance our understanding of spatiotemporal regulation of CLE perception modules in the future. In addition, the molecular mechanisms of CLE-receptor binding deserve further study. So far, protein structures have only been resolved for a handful of CLE-receptor pairs (Zhang et al. 2016; Li et al. 2017; Roman et al. 2022). Characterizing the receptor complexes for environment-regulated CLE peptides and elucidating binding affinities for closely related peptide–receptor complexes will be instrumental to better understand how environment- and CLE-specific plant responses are achieved.

Finally, we anticipate that more mechanistic studies on environment-responsive CLAVATA signaling will be conducted in crop species. This requires a thorough understanding of *CLE* diversity across plant species. Due to their small size and variable sequence apart from the conserved CLE domain, *CLE* genes are difficult to identify in genomes. Recently, computational methods have been developed that allowed the identification of a previously unknown *CLE* gene even in the well-studied model plant *Arabidopsis* and 37 additional *CLE* genes in tomato (on top of the 15 known ones) (Carbonnel et al. 2022, 2023). Applying these computational tools to other crops holds great potential to identify more peptide regulators of plant–environment interactions. Because CLAVATA signaling regulates plant responses to various environmental stimuli, including abiotic stressors and biotic interactions, fine-tuning these signaling mechanisms under field conditions may allow to create crops with enhanced resilience to environmental challenges associated with rapid climate change.

**OUTSTANDING QUESTIONS**

- Do CLE peptides induced by a single environmental stimulus perform specific or redundant functions?
- What are the upstream regulatory processes that allow a single *CLE* gene to respond to multiple environmental stimuli?
- What are the cellular- and tissue-level expression patterns of environment-responsive *CLE* genes?
- How is CLE-specific signal perception by CLAVATA-type receptors achieved?
- What are the components of the signaling cascade downstream of CLAVATA receptors?
- How do CLE peptides and CLAVATA-type receptors integrate different environmental cues?
- Are *CLE* genes conserved in plant-interacting organisms other than those reported so far?
- (How) can we apply our knowledge on CLAVATA signaling for crop improvement?

## Data Availability

There are no new data associated with this article.

## Dive Curated Terms

The following phenotypic, genotypic, and functional terms are of significance to the work described in this paper:

CLV2 Gramene: AT1G65380
CLV2 Araport: AT1G65380

rpk2 Gramene: AT3G02130
rpk2 Araport: AT3G02130

pll1 Gramene: AT2G35350
pll1 Araport: AT2G35350

CLV1 Gramene: AT1G75820
CLV1 Araport: AT1G75820

CLE12 Gramene: AT1G68795
CLE12 Araport: AT1G68795

RIC1 Gramene: AT2G33460
RIC1 Araport: AT2G33460

CLE14 Gramene: AT1G63245
CLE14 Araport: AT1G63245

CLE3 Gramene: AT1G06225
CLE3 Araport: AT1G06225

BAM1 Gramene: Solyc02g091840
BAM1 Araport: Solyc02g091840
